# Aquaporin-11: A channel protein lacking apparent transport function expressed in brain

**DOI:** 10.1186/1471-2091-7-14

**Published:** 2006-05-01

**Authors:** Daniel A Gorelick, Jeppe Praetorius, Takashi Tsunenari, Søren Nielsen, Peter Agre

**Affiliations:** 1 Department of Biological Chemistry, Johns Hopkins University School of Medicine, Baltimore, USA; 2 The Water and Salt Research Center, Institute of Anatomy, University of Aarhus, Aarhus, Denmark; 3 Department of Neuroscience, Johns Hopkins University School of Medicine, Baltimore, USA; 4 Department of Embryology, Carnegie Institution of Washington, 3520 San Martin Drive, Baltimore, MD 21218, USA

## Abstract

**Background:**

The aquaporins are a family of integral membrane proteins composed of two subfamilies: the orthodox aquaporins, which transport only water, and the aquaglyceroporins, which transport glycerol, urea, or other small solutes. Two recently described aquaporins, numbers 11 and 12, appear to be more distantly related to the other mammalian aquaporins and aquaglyceroporins.

**Results:**

We report on the characterization of Aquaporin-11 (AQP11). AQP11 RNA and protein is found in multiple rat tissues, including kidney, liver, testes and brain. AQP11 has a unique distribution in brain, appearing in Purkinje cell dendrites, hippocampal neurons of CA1 and CA2, and cerebral cortical neurons. Immunofluorescent staining of Purkinje cells indicates that AQP11 is intracellular. Unlike other aquaporins, *Xenopus *oocytes expressing AQP11 in the plasma membrane failed to transport water, glycerol, urea, or ions.

**Conclusion:**

AQP11 is functionally distinct from other proteins of the aquaporin superfamily and could represent a new aquaporin subfamily. Further studies are necessary to elucidate the role of AQP11 in the brain.

## Background

The aquaporins (AQP), also known as the major intrinsic protein superfamily (MIP), are a family of integral membrane proteins. In mammals, 13 aquaporins were described (AQP0 – AQP12). There are two subfamilies: the aquaporins, which transport only water, and the aquaglyceroporins, which transport glycerol, urea, and other small solutes in addition to water [1]. An exception is AQP6, which is permeated by anions [2]. Aquaporins and aquaglyceroporins are present throughout the body, including in the plasma membrane of renal epithelia, brain astrocytes, and red blood cells [3].

Here we focus on AQP11, a protein with lower homology to previously characterized aquaporins and aquaglyceroporins. AQP11 was previously described in mice [4], although the authors were unable to measure solute transport. We aimed to establish the tissue distribution of rat AQP11 and to identify solutes transported by rat and human AQP11.

## Results and discussion

### Cloning and sequence analysis of AQP11

Searching the human genome using Psi-BLAST, we found two genes with sequences related to members of the aquaporin gene family (also known as the MIP superfamily). Both were also present in rat and mouse genomes. These genes were previously annotated as AQP11 and AQP12 (GenBank: NP_766627 and GenBank: NP_945349). A search for conserved domains [5] revealed a significant alignment with MIP domain, a feature of all aquaporins and aquaglyceroporins. Mouse AQP12 is expressed in pancreas; transport studies were inconclusive because tagged AQP12 failed to traffic to the surface of *Xenopus *oocytes [6]. During preparation of this manuscript, Morishita and colleagues published a report on mouse AQP11; transport studies were also inconclusive because tagged AQP11 failed to traffic to the surface of *Xenopus *oocytes [4].

**Figure 1:**
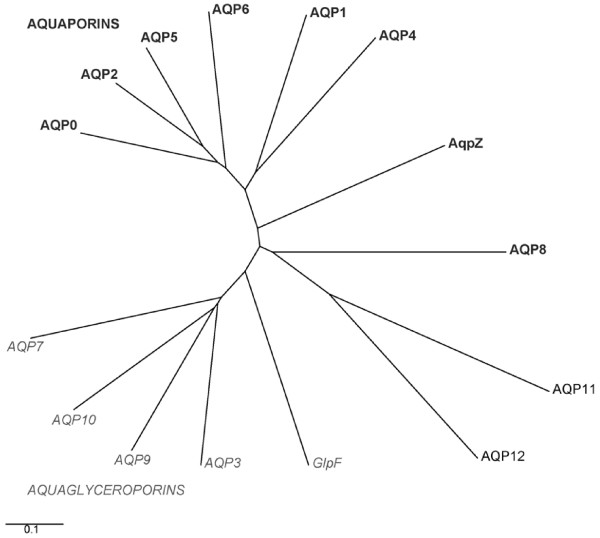
**Phylogenetic tree of the human Aquaporin gene family**. Water permeable aquaporins are shown in bold (AQP0, 1, 2, 4, 5, 6, 8, AqpZ). Glycerol permeable aquaglyceroporins are in italics (AQP3, 7, 9, 10, GlpF). *E. coli *homologues are AqpZ and GlpF. The unclassified subfamily comprising AQP11 and 12 is on the bottom right. The scale bar represents genetic distance between homologues.

**Figure 2:**
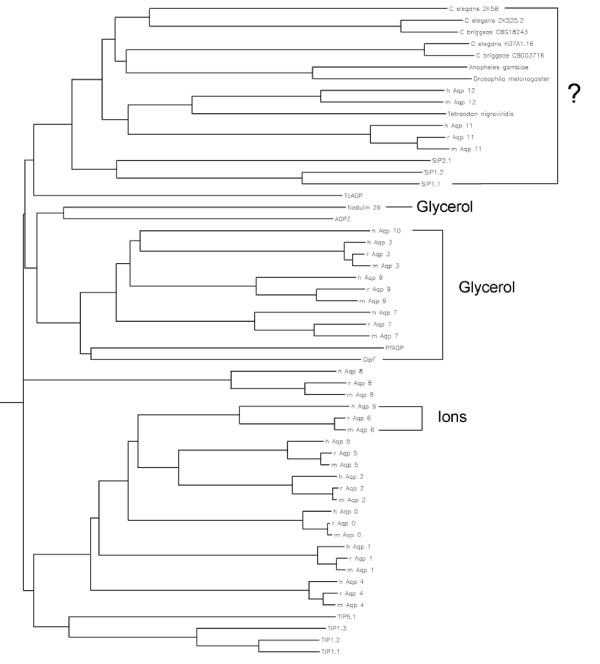
**Major Intrinsic Protein Superfamily**. Phylip rooted phylogenetic tree of aquaporin proteins from diverse species. Shown are aquaporins from mouse (m), rat (r), and human (h); from plants *Arabidopsis thaliana *(SIPs, TIPs) and soybean *Glycine max *(Nodulin 26); from bacteria *E. coli *GlpF and AqpZ; from intracellular parasites *Trypanosoma cruzi *(TcAQP) and *Plasmodium falciparum *(PfAQP); from nematodes *C. elegans *and *C. briggsae*; from fruitfly *D. melanogaster *and mosquito *A. gambiae*; from pufferfish *T. nigroviridis*. Aquaporins that transport glycerol or ions, in addition to water, are labeled by brackets on the right. Aquaporins hat transport only water are unlabelled. Aquaporins for which there is no known solute are labeled with a question mark. In many cases only one or two of the mammalian aquaporins in a specific group have been characterized. For example, TIP1.1 is a water-selective channel, but TIP5.1 has not been characterized; human and rat AQP6 are ion and water channels, but mouse AQP6 has not been characterized.

Using PCR, we amplified human AQP11 from colon cDNA, and mouse and rat AQP11 from testes cDNA. Human AQP11 is 91% similar and 82% identical to rat AQP11, and 92% similar and 83% identical to mouse AQP11 (mouse and rat AQP11 are 98% similar and 93% identical). AQP11 is 24–33% similar (28% on average), and approximately 10% identical, to the previously characterized mammalian aquaporins (AQP0 – 10), but 41% similar and 23% identical to AQP12. AQP11 is most similar to AQP12, and least similar to AQP4 and AQP7 (24%). AQP11 and AQP12 are similar to non-mammalian aquaporins present in plant *Arabidopsis thaliana *(SIPs, ~28%), puffer-fish *Tetraodon nigroviridis *(44%), mosquito *Anopheles gambiae *and fruitfly *Drosophila melanogaster *(40%), and nematodes *Caenorhabditis elegans *and *C. briggsae *(~37%). In addition to mouse and rat, human AQP11 has homologous putative genes in the dingo *Canis familiaris *(gene LOC476798) and in the chicken *Gallus gallus *(gene LOC426725). Sequence alignments and analysis of the phylogenetic tree suggest that AQP11 and 12 are distinct from the known mammalian aquaporins and aquaglyceroporins (Figure 1) and may comprise a new MIP subfamily (Figure 2).

**Figure 3:**
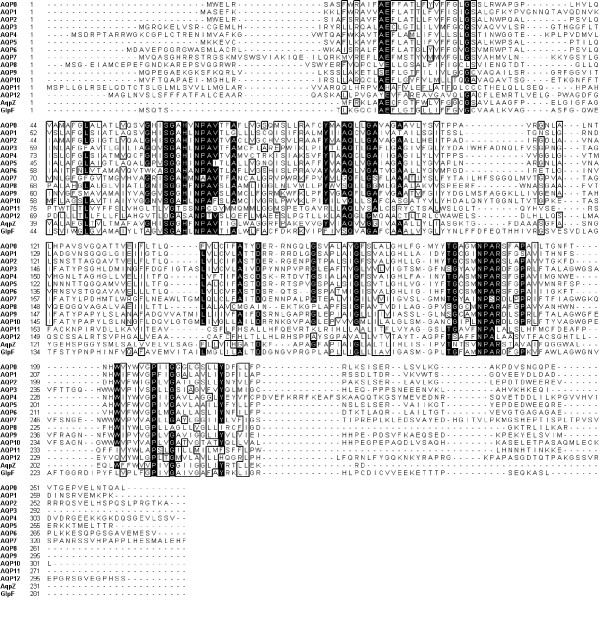
**Sequence alignment of human Aquaporins**. Clustal alignment of human aquaporins and *E. coli *AqpZ and GlpF proteins. Identical residues are highlighted in black, residues with at least 70% similarity are boxed.

Compared to other mammalian aquaporins, AQP11 has several unique attributes (Figure 3). The two Asn-Pro-Ala motifs of the aquaporin superfamily are present, although the first contains a cysteine instead of an alanine. Following the second Asn-Pro-Ala, AQP11 has a leucine, where all other mammalian aquaporins have an arginine. Based on the high-resolution structures of AQP0, AQP1, GlpF, and AQPZ [7-10], this leucine is one of four residues which form the selectivity filter, the narrowest region of the monomeric pore. In the region of the pore leading from the constriction point to the cytoplasm, the other aquaporins have Ser-Gly-(Ala/Gly)-His. AQP11 has Val-Gly-Thr-Ser. The histidine (serine in AQP11) is conserved in all other mammalian aquaporins. Similarly, the region of the pore leading to the outside of the membrane is not conserved in AQP11. AQP11 contains a bulky, aromatic phenylalanine, where the other aquaporins have a methionine, isoleucine, leucine, or valine. These changes may have a significant impact on solute transport. Based on this sequence alignment, the polarity and size of the constriction region of AQP11 is quite different from the other aquaporins. In addition, the change in amino acids lining the pore could greatly limit the size and/or charge of solutes transported by AQP11. Because the similarity to other aquaporins for which there is a high-resolution structure is so low, we were unable to use homology modeling to predict the 3-dimensional structure of AQP11.

### AQP11 mRNA expression in rat tissues

**Figure 4:**
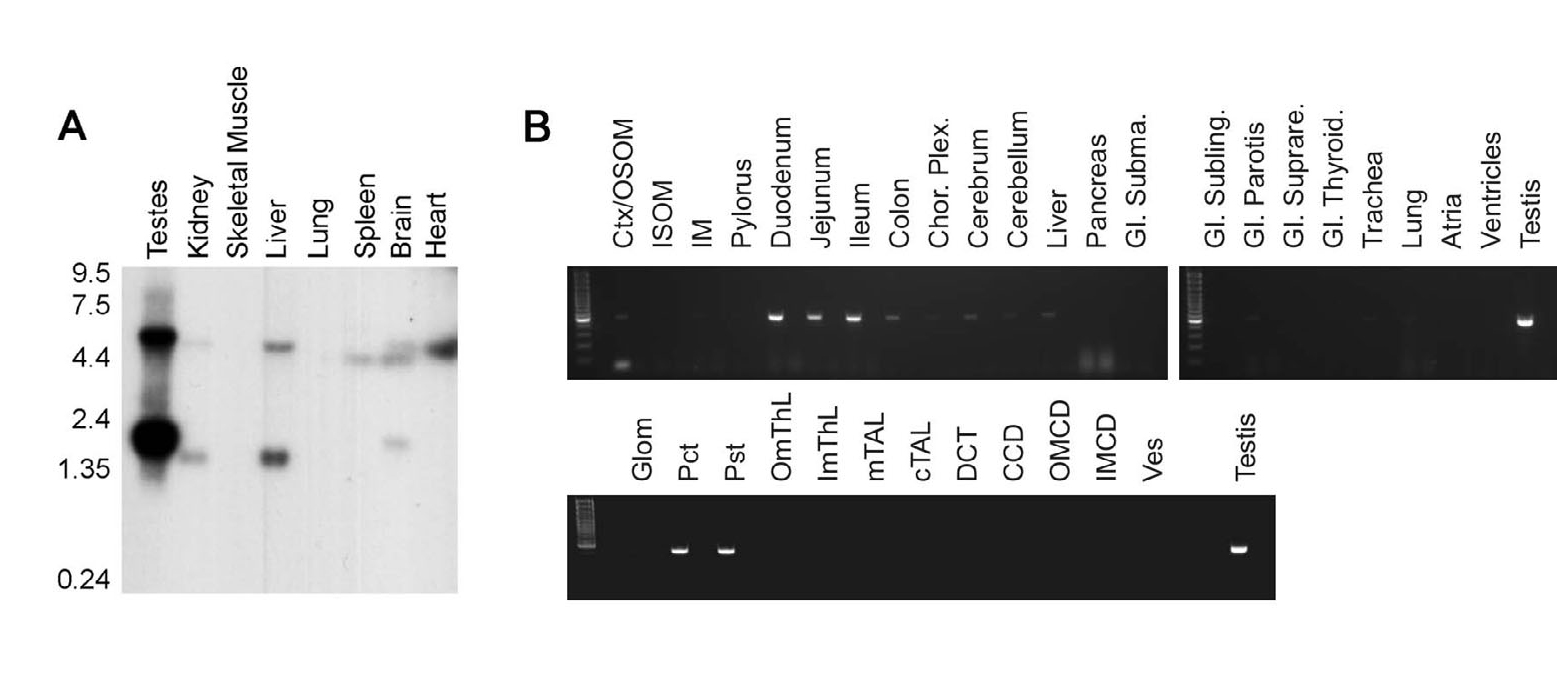
**AQP11 is expressed in multiple rat tissues**. (A) Northern blot of multiple rat tissue poly(A) RNA was probed with a portion of AQP11 mRNA. Molecular sizes are indicated in kilobases. (B) Agarose gel of AQP11 RT-PCR products from rat kidney cortex & outer stripe of outer medulla (Ctx/OSOM), inner stripe of outer medulla (ISOM), inner medulla (IM), GI tract, choroid plexus (Chor Plex), brain, liver, pancreas, submaxillary (Gl Subma), sublingual (Gl Subling), parotis (Gl parotis) and suprarenal (Gl Suprare) glands, thyroid, trachea, lung, heart and testes (upper panel) or isolated renal glomeruli (Glom), proximal convoluted and straight tubules (Pct, Pst), thin limbs of outer and inner medulla (OmThL, ImThL), medullary and cortical thick ascending limbs of Henle's loop (mTAL, cTAL), distal convoluted tubules (DCT), collecting ducts of the cortex (CCD), outer medulla (OMCD) and inner medulla (IMCD) and blood vessels (Ves) (lower panel). Molecular size markers are shown to the left; the brightest band represents 500 basepairs. Ctx/OSOM = kidney cortex & outer stripe of outer medulla; ISOM = kidney inner stripe of outer medulla; IM = kidney inner medulla.

We probed a rat multiple-tissue Northern blot for AQP11 (Figure 4A). The rat AQP11 predicted open reading frame is 816 nucleotides, coding for 271 amino acids. We found prominent bands, between 1.5 and 2 kilobases (kb), in testes, liver, kidney and brain. The bands in kidney and liver were slightly smaller than the bands in testes and brain. These tissues also had bands above 4.5 kb, as did spleen and heart.

RT-PCR analysis revealed the expression of mRNA encoding AQP11 in a range of rat tissues (Figure 4B). In agreement with our Northern blot, we found AQP11 message in brain cerebrum, in the renal cortex, and in testis. We also detected AQP11 in the intestinal tract from duodenum to ileum, and weaker expression in choroid plexus, cerebellum, colon and liver. Since this RT-PCR was not quantitative, it is impossible to infer relative message abundance. In microisolated renal structures, we found mRNA only in proximal tubules and not in glomeruli, thin limbs of the medulla, thick ascending limbs of the medulla and cortex, distal convoluted tubules, or in collecting ducts (Figure 4B, bottom panel). This is in agreement with a recent study demonstrating AQP11 immunostaining in mouse kidney cortex proximal tubule but not in distal tubule [4].

### Characterization of anti-AQP11 antibodies in cultured cells

**Figure 5:**
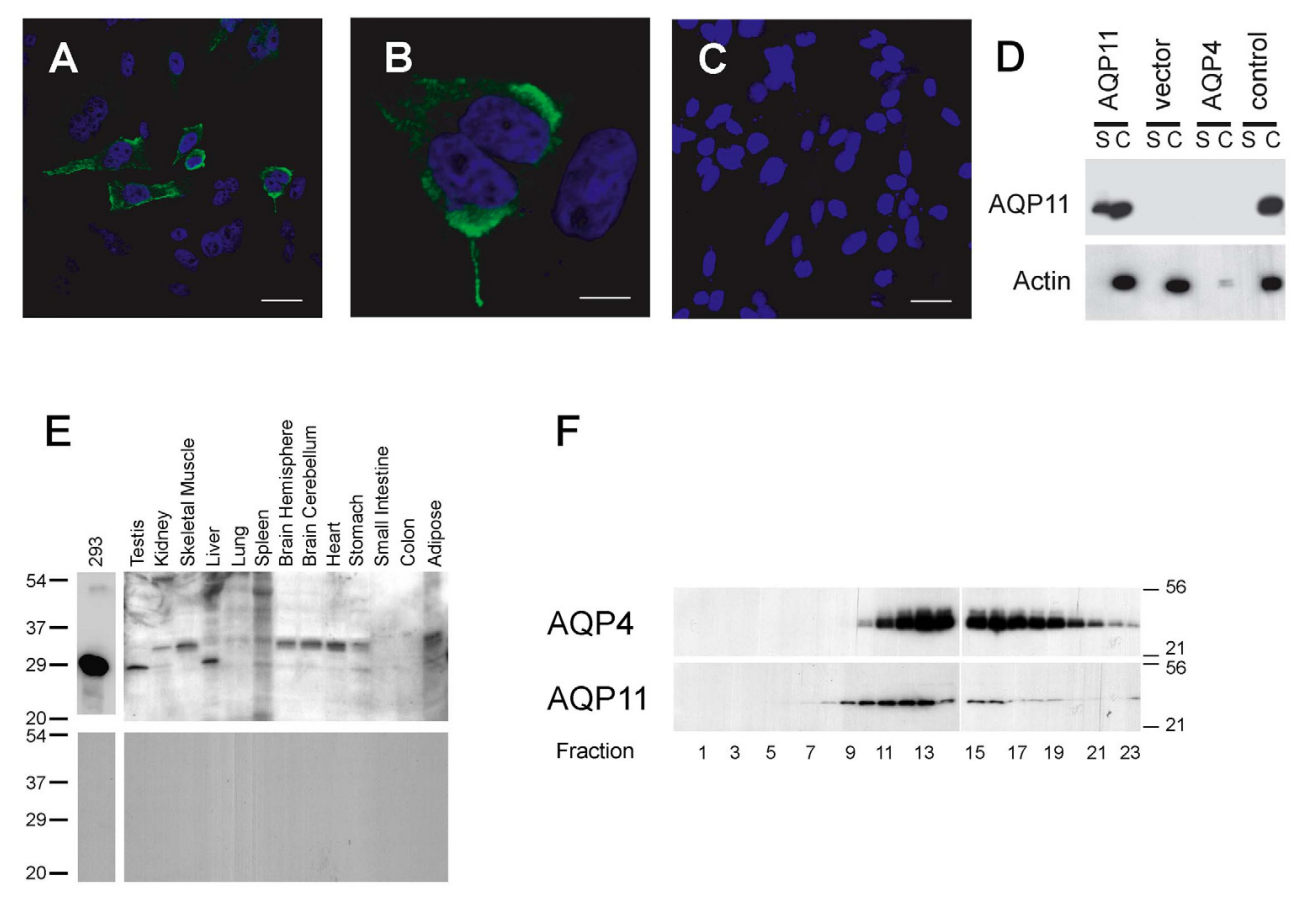
**Validation of anti-AQP11 antibody and tissue immunoblotting**. (A) – (C) Confocal images of cultured CHO cells transiently transfected with rat AQP11 (A, B) or with empty vector (C) and stained with anti-AQP11 C-term antibody. Bars represent 20 μm (A, C) and 5 μm (B). (D) CHO cells were transiently transfected with rat AQP11, empty vector, or rat AQP4 M23 and treated with membrane impermeable biotin, then precipitated with streptavidin. Surface proteins eluted from the streptavidin (S) and cytosolic proteins from the supernatant (C) were analyzed by SDS-PAGE followed by immunoblotting with antibodies against AQP11 and actin. As a control, cells transfected with AQP11 were precipitated with streptavidin but not incubated with biotin. (E) Western blot of multiple rat tissue probed with AQP11 antibody (upper panel). The bottom panel was probed with the same antibody preincubated with antigenic peptide. (F) Sucrose density sedimentation of AQP11 from rat brain. Membrane proteins from rat brain hemispheres were layered onto a 5–20% linear sucrose gradient, spun, and analyzed by SDS-PAGE followed by immunoblotting for either AQP4 or AQP11. Fraction 1 corresponds to the lightest fraction, 23 to the heaviest. Molecular size in kDa is shown.

To elucidate AQP11 protein distribution, we generated antibodies against the C-terminus and N-terminus of rat AQP11. We transiently transfected cultured Chinese Hamster Ovary cells (CHO) with rat AQP11 and stained them with our C-term antibody (Figure 5A–C). We saw predominantly intracellular staining and some staining at the plasma membrane, and no staining in cells transfected with empty vector. AQP11 immunoblots of surface biotinylated CHO cell lysates confirmed that AQP11 is present at both the plasma membrane and in the cytosol in transfected CHO cells (Figure 5D). We saw no bands in cells transfected with empty vector or in cells transfected with Aquaporin-4, demonstrating the specificity of our C-term antibody. We obtained similar results in transiently transfected MDCK and HEK293 cells using both N-term and C-term antibodies (not shown).

### AQP11 protein expression in rat tissues

Based on amino acid composition, the estimated molecular weight of rat AQP11 is 30 kDa. An anti-AQP11 C-term immunoblot of multiple rat tissue membrane proteins revealed two prominent bands: an approximately 32 kDa band in brain, kidney, heart, and skeletal muscle; and an approximately 25 kDa band in testes, kidney, and liver (Figure 5E upper panel, calculated based on migration distance vs. markers of known size; similar results were obtained with the N-term antibody, not shown). De-glycosylation (PNGase treatment) or de-phosphorylation (lambda protein phosphatase treatment) assays did not produce changes in protein migration patterns (not shown). As a control, we also probed total protein lysate from cultured HEK293 cells transfected with rat AQP11. This produced only the 25 kDa band. Both bands disappeared when the antibody was preadsorbed to the antigenic peptide (Figure 5E, lower panel). There appears to be no correlation between the transcript size (Northern blot) and the protein size (immunoblot), as the testes contain a larger band in the Northern blot (2 kb) but a smaller band in the Western blot.

Our results demonstrate rat AQP11 RNA and protein are found in testis, kidney, liver, and brain. We failed to detect AQP11 RNA in skeletal muscle and heart (Northern blot, Figure 4A), although Western blot of these tissues was positive (Figure 5E). Since RNA loading was normalized against β-actin hybridization signal (not shown), this discrepancy could be due to low levels of AQP11 RNA in these tissues.

Biochemical and structural evidence shows that many mammalian aquaporins form tetramers [7-9,11]. We subjected rat brain hemisphere membrane proteins to sucrose density ultra-centrifugation and found that AQP11 sediments in similar fractions as AQP4, a water channel known to form tetramers (Figure 5F). In agreement with previous work, we detected AQP4 in fractions 10–23, with a peak in fractions 13–15 [11]. AQP11 was found in fractions 8–19, with a peak in fractions 11–13. This distribution overlaps significantly, though not perfectly. Regardless, the first several fractions lack AQP11, and it is in these fractions where we would expect to find an AQP11 monomer. We conclude that AQP11, like AQP4, exists in rat brain as a multi-subunit oligomer – presumably a tetramer like AQP4 and other aquaporins.

### Functional expression of AQP11 in xenopus Oocytes

**Figure 6:**
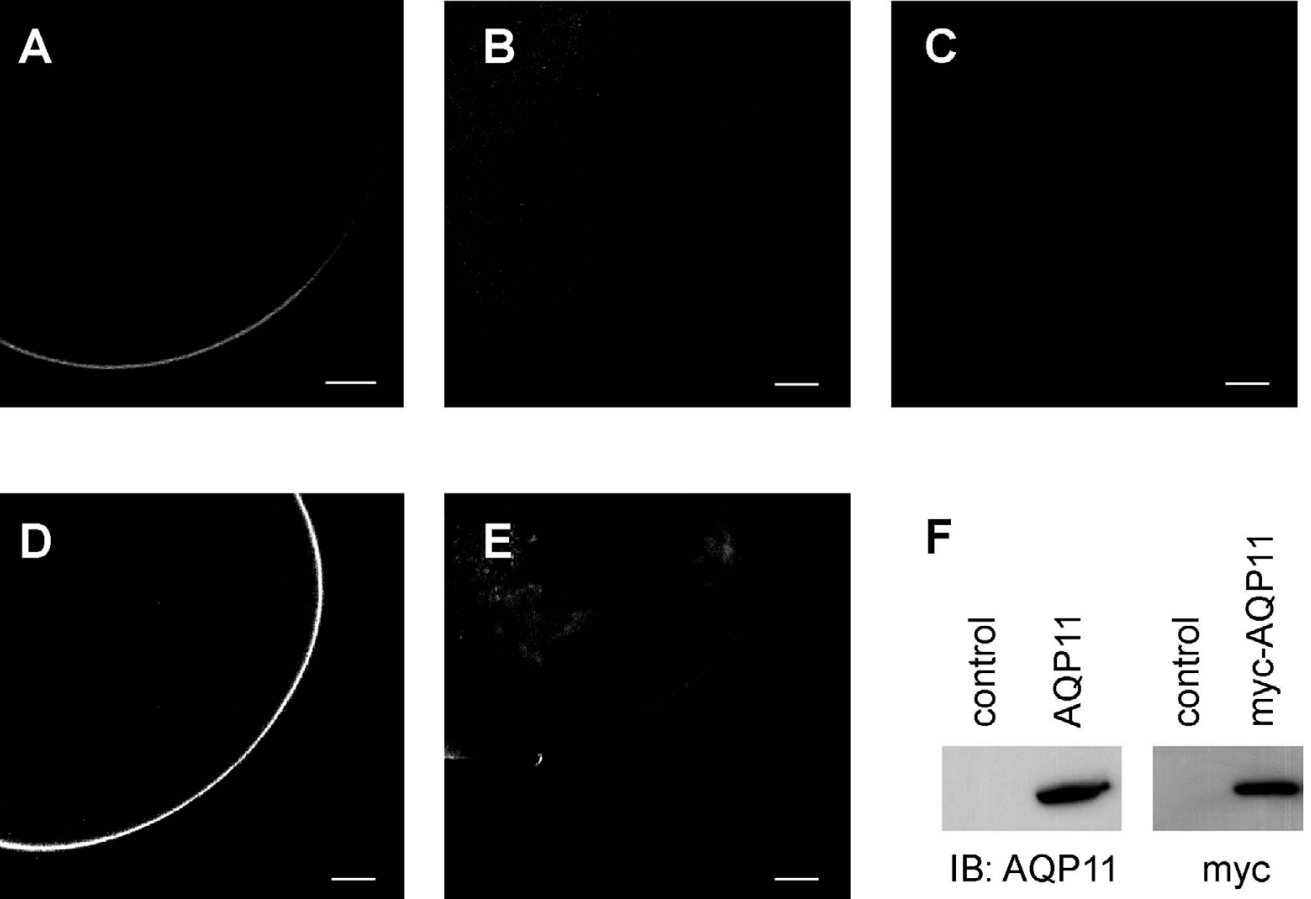
**Expression of AQP11 in *Xenopus *oocytes**. Oocytes were injected with rat AQP11 cRNA (A, B), myc-tagged human AQP11 (D), or water (C, E) and stained with an antibody against rat AQP11 (A, C), with the antibody preadsorbed to antigenic peptide (B), or with an antibody against myc (D, E). Scale bars represent 100 μm. (F) Immunoblots of total protein from oocytes injected with rat AQP11 (AQP11), myc-tagged human AQP11 (myc-AQP11) or water (control). Blots were probed with anti-AQP11 antibody or with anti-myc antibody, respectively.

We injected oocytes with human AQP11, myc-tagged human AQP11, or rat AQP11 and confirmed surface expression of the second two proteins by immunofluorescence microscopy and by immunoblot (Figure 6). Using an oocyte swelling assay, we tested AQP11-injected oocytes for transport of water, glycerol, or urea (Table 1). Compared to controls, oocytes injected with 5 ng or 25 ng of human wildtype or myc-tagged AQP11 cRNA showed no increase in the rate of water transport. Rate of water transport was significantly less than in oocytes expressing an orthodox water channel, such as human AQP1 or rat AQP2 (control = 16.88 ± 6.65; myc-human AQP11 = 15.32 ± 5.64; AQP1 = 263.92 ± 42.22). Additionally, we failed to detect transport of glycerol or urea in AQP11-injected oocytes. Rate of glycerol and urea transport was similar to control oocytes, and significantly less than in oocytes expressing rat AQP9, an aquaglyceroporin that transports glycerol and urea (glycerol: human AQP11 = 0.79 ± 1.27, AQP9 = 11.84 ± 4.69; urea: human AQP11 = 0.03 ± 0.24, AQP9 = 11.14 ± 5.17).

**Table 1:** AQP11 injected oocytes show no increase in water, glycerol or urea transport, and no increase in ion conductance at pH 7.5.

	Water Transport	Glycerol Transport	Urea Transport	Ion Conductance
control	16.88 ± 6.65	1.53 ± 2.31	-2.04 ± 7.05	2.8 ± 0.6
hAQP1	263.92 ± 42.22			
myc-hAQP11	15.32 ± 5.64	1.67 ± 3.25	0.96 ± 2.78	1.9 ± 0.1
rAQP9		21.12 ± 7.57	16.85 ± 9.33	
control Hg		2.18 ± 3.76	0.74 ± 1.91	
rAQP9 Hg		-1.44 ± 1.06	0.53 ± 2.11	
myc-hAQP11 Hg		-0.52 ± 1.12	2.51 ± 2.88	
				
control	20.63 ± 9.94			3.5 ± 1.1
rAQP2	142.91 ± 39.21			
25 ng hAQP11	11.89 ± 2.32			
hAQP11	11.26 ± 4.77	0.79 ± 1.27	0.03 ± 0.24	5.3 ± 2.5
rAQP9		11.84 ± 4.69	11.14 ± 5.17	
control Hg	28.39 ± 13.58			
rAQP2 Hg	54.11 ± 21.39			
hAQP11 Hg	29.62 ± 23.90			
rAQP11				4.4 ± 0.7
rAQP6				10.0 ± 3.4
				
control	10.86 ± 14.23	5.74 ± 6.61	-0.45 ± 2.21	
hAQP1	196.61 ± 24.52			
rAQP11	17.64 ± 10.89	5.91 ± 8.24	0.005 ± 1.14	
rAQP9			3.11 ± 2.64	

*Xenopus *oocytes were injected with 50 nl water (control), or with 5 ng human AQP1 (hAQP1), rat AQP2 (rAQP2), rat AQP9 (rAQP9), human AQP11 (hAQP11), myc-tagged human AQP11 (myc-hAQP11), or with 25 ng human AQP11 cRNA and tested for water, glycerol, urea, and ion transport. Some oocytes were pre-incubated for 5 minutes with 0.1 mM HgCl_2 _(Hg). Units for water transport are μm/sec, for glycerol and urea transport 10^-6 ^cm/sec, and for ion conductance μA/V. Data represent mean ± SD for 6–10 oocytes, except for ion conductance which is mean ± SE for 3–5 oocytes.

Solute transport through other aquaporins, such as AQP3 and AQP6, is affected by pH [2,12]. Lack of solute transport by AQP11-injected oocytes, however, was unaffected by raising the pH to 8.5, or by lowering the pH to 6 or 4.5 (Table 2). Incubating the oocytes with HgCl_2 _did not alter the water, glycerol, or urea permeability of AQP11-injected oocytes, although it did inhibit water transport in AQP2-injected oocytes and glycerol and urea transport in AQP9-injected oocytes (Table 1). We also failed to see a change in ion conductance in oocytes expressing AQP11 (Table 1). Oocytes injected with AQP6, however, showed an appreciable increase in conductance compared to water injected oocytes. At pH 4.5, AQP11 injected oocytes showed no significant difference in ion conductance versus water injected control oocytes (Table 2). Similar results occurred at pH 8.5 (Table 2), whereas AQP6 injected oocytes displayed conductance values almost five times higher than oocytes injected with rat or human AQP11 or with water (control = 4.7 ± 1.4, human AQP11 = 5.7 ± 2.8, rat AQP11 = 5.1 ± 1.1, AQP6 = 23 ± 2.6).

**Table 2:** AQP11 injected oocytes show no increase in water, glycerol, or urea transport at pH 4.5, 6.5, or 8.5, and no increase in ion conductance at pH 4.5 or 8.5.

	Water Transport	Glycerol Transport	Urea Transport	Ion Conductance
pH 4.5				
control	8.87 ± 3.76			2.2 ± 0.2
hAQP1	234.06 ± 23.52	-4.41 ± 1.91	4.77 ± 4.42	
myc-hAQP11	8.57 ± 4.19	-0.94 ± 1.63	4.23 ± 4.82	1.7 ± 0.2
				
pH 6				
control	19.13 ± 8.2	0.63 ± 1.83	3.61 ± 5.2	
hAQP1	209.22 ± 85.63			
myc-hAQP11	27.09 ± 29.76	0.24 ± 2.57	1.17 ± 4.23	
				
pH 8.5				
control	31.23 ± 11.83			4.7 ± 1.4
rAQP4	196.16 ± 56.43	9.26 ± 3.24	-3.65 ± 5.11	
hAQP11	33.92 ± 37.23			5.7 ± 2.8
rAQP11				5.1 ± 1.1
rAQP6				23 ± 2.6
25 ng hAQP11	20.85 ± 6.69	4.21 ± 5.18	-0.32 ± 1.97	

*Xenopus *oocytes were injected with 50 nl water (control), or with 5 ng human AQP1 (hAQP1), rat AQP2 (rAQP2), rat AQP4 M23 (rAQP4), rat AQP6 (rAQP6), rat AQP11 (rAQP11), human AQP11 (hAQP11), myc-tagged human AQP11 (myc-hAQP11), or with 25 ng human AQP11 cRNA and tested for water, glycerol, or urea transport or ion conductance at indicated pH. Units for water transport are μm/sec, for glycerol and urea transport 10^-6 ^cm/sec, for ion conductance μA/V. Data represent mean ± SD for 6–10 oocytes, except for ion conductance which is mean ± SE for 3–5 oocytes.

Based on these oocyte studies, we conclude that under these conditions AQP11 does not transport water, glycerol, urea, or ions. It is possible that a putative AQP11 channel is closed unless a ligand or other regulatory molecule is present. It is also possible that AQP11 does not act as a solute channel in vivo. Like AQP0, a poor water channel that forms membrane junctions in the lens of the eye [9,13], the role of AQP11 may be primarily structural. It is also possible that AQP11 transports a different, untested solute, such as ammonium, CO_2_, or NO_2_. Finally, it is possible that AQP11 is not functional when expressed in oocytes. If true, this would be the first aquaporin that fails to function when expressed in oocytes.

A previous study reported that exogenous mouse AQP11 was not targeted to the plasma membrane in oocytes [4]. This conflicts with our observations using rat AQP11. This discrepancy could be due to the amino acid differences between rat and mouse AQP11. Alternatively, it could be because the previous study utilized AQP11 tagged with GFP or V5. These tags may alter AQP11 localization in oocytes.

### AQP11 in rat brain

**Figure 7:**
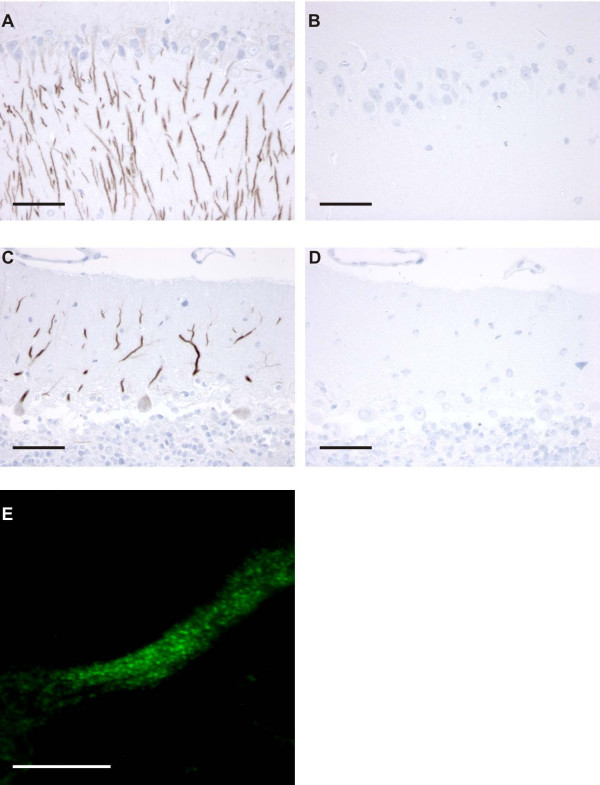
**Localization of AQP11 in rat cerebellum and hippocampus**. Semi-thin sections of rat brain were stained with an anti-N-terminal AQP11 antibody. (A) The CA1 and CA2 regions of the hippocampus displayed a filamentous labeling pattern. (B) Preabsorbing the antibody with the immunizing peptide prevented labeling in an adjacent section. (C) The dendrites of Purkinje cells showed strong AQP11 immunoreacivity which was absent after peptide preabsorption (D). Bars indicate 50 μm. (E) Immunofluorescence confocal micrograph showing punctuate labeling inside a Purkinje dendrite. Bars indicate 10 μm.

To further elucidate the location of AQP11 in rat brain, we stained rat brain sections with AQP11 N-term antibody. We found staining primarily in the cerebellum, hippocampus, and cerebral cortex (Figure 7). In the cerebellum, AQP11 immunoreactivity was associated with the dendrites of Purkinje cells. Immunofluorescence microscopy revealed punctate labeling, indicative of intracellular localization (Figure 7E). In the hippocampus, AQP11 positive neurons were found in the CA1 and CA2. In cerebral cortex, staining was absent in cortical layer I, but present in layers II-VI. We failed to see strong staining in other areas of the brain, such as in the thalamus or near the ventricles. Similar staining was obtained with anti-C-terminal AQP11 antibodies (not shown). Future studies using electron miscroscopy are necessary to more definitively assess the cellular localization of AQP11.

A previous study demonstrated AQP11 protein expression in mouse kidney [4]; using our AQP11 antibodies we were unable to see staining in rat kidney (not shown). The authors of the previous study did not stain brain sections, but they did note that mice lacking AQP11 began dying 15 days after birth, with renal failure and polycystic kidneys [4]. One explanation for the discrepancy might be due to differences in AQP11 localization in mouse versus rat kidney. In rat kidney, perhaps the termini of AQP11 are not accessible for antibody interaction.

AQP11 localization in the brain is different from the other brain aquaporins. AQP4 is restricted to astrocytes throughout the brain, while AQP1 is present in the choroid plexus [14]. AQP9 is present in astrocytes [15] as well as in a population of catecholaminergic neurons [16]. The apparent intracellular localization of AQP11 in Purkinje cells is unusual. Only AQP6 is predominantly intracellular in vivo. The unique distribution and the apparent lack of water/solute transport by AQP11 may predict a unique function.

## Conclusion

We describe rat AQP11, an aquaporin with lower similarity to known mammalian aquaporins and aquaglyceroporins. AQP11, along with AQP12, may comprise a new aquaporin subfamily. Unlike other aquaporins, we were unable to demonstrate transport of water, glycerol, urea, or ions by AQP11. This is likely due to putative pore-lining residues that are not conserved between AQP11 and other mammalian aquaporins. AQP11 is expressed in a variety of rat tissues. In the brain, AQP11 is found in Purkinje cells, hippocampal neurons of CA1 and CA2, and cerebral cortical neurons. Future studies are necessary to elucidate the physiological role of AQP11 in the brain.

## Methods

### Cloning and sequence alignment

AQP11 was found using a Psi-BLAST search of GenBank with the BLOSUM62 matrix. Multiple sequence alignment and phylogenetic tree generation was performed using ClustalX 1.81 and the Gonnett series matrix [17]. The open reading frame of human AQP11 was amplified from human colon cDNA library (BD Biosciences, San Jose, CA) using primers 5'AQP11A (5'-GGCGACGAATTCATGTCGCCGCTGCTGGGGCTC-3') and 3'AQP11 (5'-TCTTTGTCTAGATTATTCCTTTTTATTAATTGTATG-3'). The open reading frame of rat AQP11 was cloned from testicle poly(A) RNA (Ambion, Austin, TX) using OneStep RT-PCR (Qiagen, Valencia, CA) and primers rQ11EcoRI (5'-ATGCGAATTCATGTCCGCGCTACTGGGGCT-3') and rQ11XbaI (5'-ATCGTCTAGATCATTCCTTCTTGTTACTCA-3'). Rat AQP11 was cloned into pcDNA3.1Zeo (Invitrogen, Carlsbad, CA) or pcDNA3.1Zeo-myc for expression in mammalian cells, and into pXBG for transcription of cRNA [18], using standard PCR cloning techniques. All cloning was performed by introducing EcoRI and XbaI restriction enzyme sites (underlined) into the primers. All clones were verified by both restriction digest and sequencing from both ends of the multiple cloning site.

### RNA purification and RT-PCR

Adult male Wistar rats (250–400 g) from M&B (Eiby, Denmark) had free access to water and pelleted food. The animals were anesthetized by isoflurane inhalation before removing various organs and sacrificing the animals. Organs were rinsed in a 4°C saline solution, and kidneys were further divided into samples of cortex (Ctx, with outer stripe of outer medulla), inner stripe of outer medulla (ISOM), and inner medulla (IM). The different segments of the renal tubular system were isolated by an enzymatic method. After perfusing the renal artery with cold saline, kidneys were removed and cut into thin slices. Ctx and IM were separated from ISOM using fine razor blades and each incubated separately 30 min at 37°C, in conical flasks containing 1 mg/ml collagenase A (Sigma, St. Louis, MO) and 2 mg/ml Dispase (Invitrogen, Taastrup, DK) in oxygenated K+/gluconate solution (in mM: 10.0 Na+, 140.0 K+, 10.0 Ca2+, 1.0 Mg2+, 32.0 Cl-, 140.0 Gluconate-, 10.0 HEPES, 10.0 Sucrose, pH 7.4) while gently shaking at 80 RPM. After wash, the tissues were agitated mildly in enzyme-free chilled buffer, and individual tubules collected by suction into loading-pipettes under a dissection microscope. For inclusion into the study, proximal convoluted tubules (PCT), proximal straight tubules (PST), and thin descending limbs of the outer medulla (OMThL) were all AQP1 positive and PCT was NBCe1 positive, inner medullary thin limbs (IMThL) were AQP1 and CLC-2 positive, medullary thick ascending limbs (mTAL) and cortical thick ascending limbs (cTAL) were NKCC2 positive, distal convoluted tulbules (DCT) were TSC positive, and collecting ducts from the cortex (CCD), outer medulla (OMCD), and the inner medulla (IMCD) were all AQP2 positive by RT-PCR.

Total RNA was purified from rat tissues using RNeasy Mini Kit (Qiagen). After DNAse treatment (RQ DNaseI, Promega, Ramcon, Denmark), total RNA was reverse transcribed using Superscript II (Life Technologies, Tåstrup, Denmark). PCR was performed for 30 cycles using sequence specific sense (5'-ATCCCTAGCGGTGAGGGAAC-3') and antisense oligonucleotide primers (5'-CACTGACTTCGTTTGAGTCTTTGG-3') with HotStarTaq Master Mix (Qiagen). Negative PCR controls included omission of reverse transcriptase or omission of cDNA. PCR products were subjected to agarose gel electrophoresis (2% agarose) and photographed under ultraviolet illumination. Kidney cortex PCR products were sequenced to confirm specificity (Lark Technologies, Essex, UK).

### Northern blot

Using PCR, base pairs 224–615 of the rat AQP11 open reading frame was subcloned into pSPT19 (Roche, Indianapolis, IN). Vector was linearized using EcoRI (New England Biolabs, Ipswich, MA) and transcription was performed using the Strip EZ RNA kit (Ambion) using T7 Polymerase and [α-^32^P]UTP (Amersham, Buckinghamshire, England) according to the manufacterer's protocol. Rat Multi-Tissue Northern Blot (Clontech, Mountain View, CA) was prehybridized for 1.5 hours at 68° in ULTRAhyb, then probed using 10^6 ^cpm/ml probe at 68° overnight in a rotating oven. Blot was washed twice for 20 minutes at room temperature using low stringency wash solution, and twice for 20 minutes at 68° using high stringency wash solution. Hybridization and wash solutions were from NorthernMax kit (Ambion).

### Aquaporin-11 antibodies

Polyclonal AQP11 antibodies were raised in rabbit (Antibodies Inc., Davis, CA) against a peptide corresponding to amino acids 259 – 271 (C-term) or amino acids 1 to 14 (N-term) of rat AQP11 (NH_2_-(C)PWLHNNQLSNKKE-COOH or NH2-MSALLGLPPEVQDT(C)-COOH, synthesized by Lofstrand, Gaithersburg, MD). Cysteines (in brackets) were added to ease the affinity purification using a column bound antigenic peptide (Pierce, Rockford, IL).

### Western blot

#### Rat tissue

Protocols were approved by the Johns Hopkins University School of Medicine Animal Care and Use Committee. 6 month old male Sprague-Dawley rats were sacrificed by asphyxiation using CO_2_. Tissues were harvested and placed in PBS plus Complete Protease Inhibitor (Roche) on ice, homogenized for 20 strokes in a drill press, then spun at 1000 g for 10 minutes. All centrifugation steps were performed at 4°. Supernatant was spun at 200,000 g for 1 hour. Membrane pellet was resuspended in 50 mM Tris-HCl pH 6.8, 3% SDS and solubilized at 37° for 1 hour. Protein concentration was determined using BCA Protein Assay (Pierce). Protein was diluted to contain 10% glycerol, 1% bromophenol blue and 1% 2-mercaptoethanol. 30 μg membrane protein was run on 12% SDS-PAGE, transferred to Immun-Blot PVDF membrane (BioRad), and probed overnight at 4° with rabbit anti-rat AQP11 antibody diluted 1:1000 in 4% BSA, 0.05% Tween-20, 1 mM NaN_3 _in PBS. Blots were washed and probed with donkey anti-rabbit HRP-conjugated secondary antibody (Amersham) for 1 hour at room temperature and visualized using ECL Plus (Amersham). When noted, blots were stripped in 100 mM 2-mercaptoethanol, 2% SDS, 62.5 mM Tris-HCl pH 6.7 at 50° for 30 minutes.

#### Cultured cells

Transient tranfections were performed using LipofectAMINE 2000 according to the manufacturer's protocol (Invitrogen). Two days following transfection, cells were homogenized in PBS with protease inhibitor as above. Membrane proteins were harvested, solubilized, and subjected to SDS-PAGE followed by immunoblotting as above.

#### Xenopus Oocytes

Three days after injection 10 oocytes per condition were mixed with 150 μl of 7.5 mM sodium phosphate and Complete Protease Inhibitor (Roche). Oocytes were lysed by vigorous pipetting, then spun at 3000 rpm in a tabletop centrifuge for 5 minutes at 4°. Supernatant was combined with sample buffer such that the final concentration was 3% SDS, 2% 2-mercaptoethanol, 50 mM Tris-HCl pH 6.8, 10% glycerol, 1% bromophenol blue and incubated for 30 minutes at 37°. 10 μl was subjected to SDS-PAGE followed by immunoblotting as above.

### Sucrose density ultracentrifugation

One rat brain hemisphere (from 7–8 week old Sprague-Dawley rat, Pel-Freez Biologicals, Rogers, AR) membrane protein preparation was solubilized in 1 ml 4% deoxycholate (DOC) in PBS for 1 hour at 37°. 300 μl was combined with 100 μl 20% sucrose and layered onto a 5–20% linear sucrose gradient in 0.2% DOC in PBS. Sample was spun at 139000 g for 16 hours at 10°. 200 μl fractions were carefully aspirated from top to bottom, subjected to SDS-PAGE and immunoblotted with antibodies to AQP4 (Alpha Diagnostic International, San Antonio, TX) or to AQP11 C-term.

### Solute Transport and ion conductance of Oocytes

Oocytes from *Xenopus laevis *frogs were harvested as described [18]. cRNA was transcribed as described [18]. Water, glycerol, and urea transport was measured as described, 2–3 days after injection [19].

Whole-cell currents of oocytes were measured two days after injection using a standard two-electrode voltage clamp amplifier (OC-725, Warner Instruments, Hamden, CT) with micropipettes of 0.5-3-MΩ resistance filled with 3 M KCl. The recordings were performed in solution containing 100 mM NaCl, 2 mM KCl, 1 mM MgCl_2_, 5 mM Na-HEPES (pH 7.5). Current and voltage data were acquired and analyzed with DIGIDATA 1200 and pClamp 6 software (Axon Instruments, Union City, CA). The membrane potential was held at -50 mV and stepped to values between -100 mV to +60 mV for 300 ms. Conductances were calculated from slopes of Current-Voltage curves between -80 mV and +20 mV. Only healthy oocytes with resting potentials of ≤-20 mV were used.

### Immunocytochemistry

#### Oocytes

2–3 days after injection, *Xenopus *oocytes were fixed in 4% paraformaldehyde (Electron Microscopy Sciences, Hatfield, PA) in PBS for 1 hour at room temperature. Oocytes were washed several times in PBS, then fixed in methanol overnight at -20°. Oocytes were washed in PBS, then incubated in 100 mM NaBH_4 _for 2 hours or overnight at room temperature. Oocytes were bisected using a razor blade, washed in PBS, then incubated overnight at 4° with rabbit anti-rat AQP11 antibody diluted 1:200 in 2% BSA in PBS. For peptide control, antibody was pre-incubated with 25 μg antigenic peptide in 1 ml for 30 minutes on ice. Oocytes were washed in PBS, then incubated overnight at 4° with donkey anti-rabbit Alexa 488 secondary antibody (Invitrogen) diluted 1:1000 in 2% BSA in PBS. Oocytes were washed in PBS, then mounted on medium thick hanging drop slides (Fisher Scientific, Hampton, NH) using Fluoromount-G (SouthernBiotech, Birmingham, Alabama). All slides were examined using an LSM510 confocal microscope (Carl Zeiss, Thornwood, NJ).

#### MDCK, HEK293 and CHO cells

Culture cells were split onto culture slides (poly-D-lysine coated for *HEK293 *cells) and transfected as above. Two days after transfection, cells were washed with PBS and fixed in 4% paraformaldehyde in PBS for 10–20 minutes at room temperature. Cells were blocked and permeabilized in 3% BSA, 1% donkey serum (Sigma), 0.1% Triton X-100 in PBS for 1 hr at room temperature, and incubated overnight at 4° with rabbit anti-rat AQP11 antibody. After wash, cells were incubated at room temperature with donkey anti-rabbit Alexa 488 secondary antibody, washed extensively in PBS, and mounted with anti-fade reagent. All antibodies were diluted in blocking solution lacking Triton X-100.

### Immunohistochemistry

Rat brains were fixed by perfusion via the left heart ventricle, with 4% paraformaldehyde in 0.1 M cacodylate buffer, pH 7.4. The tissues were dehydrated, embedded in paraffin, and 2-μm sections were cut using a rotary microtome (Leica, Heidelberg, Germany). The sections were dewaxed, rehydrated, and endogenous peroxidase was blocked by 0.5% H_2_O_2 _in absolute methanol. The sections were boiled in 10 mM Tris, pH 9, supplemented with 0.5 mM EGTA, and then incubated with 50 mM NH_4_Cl and blocked in PBS supplemented with 1% BSA, 0.05% saponin, and 0.2% gelatin. The sections were incubated overnight at 4°C with the primary antibodies diluted in PBS supplemented with 0.1% BSA and 0.3% Triton X-100.

For brightfield microscopy, the sections were incubated with horseradish peroxidase-conjugated goat anti-rabbit IgG (Dako, Glostrup, Denmark) in PBS with BSA and Triton X-100. The staining was visualized by 0.05% 3,3'diaminobenzidine tetrahydrochloride dissolved in PBS with 0.1% H_2_O_2_. Mayer's hematoxylin was used for counterstaining, and the sections were dehydrated in graded alcohol and xylene and mounted in hydrophobic Eukitt mounting medium (O. Kindler, Freiburg, Germany). For fluorescence microscopy, AQP11 labeling was visualized using an Alexa 488 conjugated goat anti-rabbit secondary antibody (Invitrogen). Sections were mounted with a coverslip in Glycergel Antifade Medium (Dako).

## Abbreviations

Aquaporin (AQP), Chinese Hamster Ovary (CHO), Madine-Darby Canine Kidney cell (MDCK), Human Embryonic Kidney (HEK)

## Authors' contributions

DG and PA conceived and designed the study and drafted the manuscript. DG performed the sequence analysis, Northern blot, cell culture, and oocyte solute transport studies. JP performed the RT-PCR, immunohistochemistry and cell culture studies and helped draft the manuscript. TT performed the ion conductance measurements. JP and SN provided the affinity purified AQP11 antibodies. All authors read and approved the final manuscript.
